# The bio-interactions between plants, insecticides and fertilizers: an innovative approach for the research of xenobiotic substances

**DOI:** 10.1007/s13659-022-00360-1

**Published:** 2022-12-01

**Authors:** Frédéric Darriet

**Affiliations:** Institute of Research for Development (IRD delegation Occitanie), Maladies Infectieuses et Vecteurs, Écologie, Génétique, Évolution et Contrôle (MIVEGEC), Université de Montpellier, IRD, CNRS, BP 64501, 911 Avenue Agropolis, 34394 Montpellier Cedex 5, France

**Keywords:** Chili pepper plant, Azadirachtin bio-insecticide, NPK fertilizer, Capsaicinoid compounds, Xenobiotic substance, *Aedes albopictus*, Innovative research

## Abstract

In this experiment carried out on Caribbean chili pepper plants (*Capsicum chinensis*), the bio-insecticide azadirachtin in combination with an NPK fertilizer proved to have a greater lethal impact on the larvae of *Aedes albopictus* than each substance on its own. This synergistic effect is noticeably important when both inputs are sprayed directly on the leaves of the plant (foliar application). While the plants treated with azadirachtin or NPK alone cause a 33.6% and 36.4% mortality respectively of the *Ae. albopictus* larvae, the combination of the two inputs induces a 74.4% mortality on the mosquito larvae. To account for this synergistic effect phenomenon inside the plant, the azadirachtin + NPK combination most likely interacts with the capsaicinoid compounds naturally produced by the plant. Not only does this study carried out on azadirachtin reveal major results but the methodology itself offers a most interesting approach on how to boost the agricultural inputs within the plants. As a matter of fact, this research axis demands developing since the control of pests harmful to men has been dramatically lacking insecticide molecules acting on new targets over the past three decades.

**Graphical Abstract:**

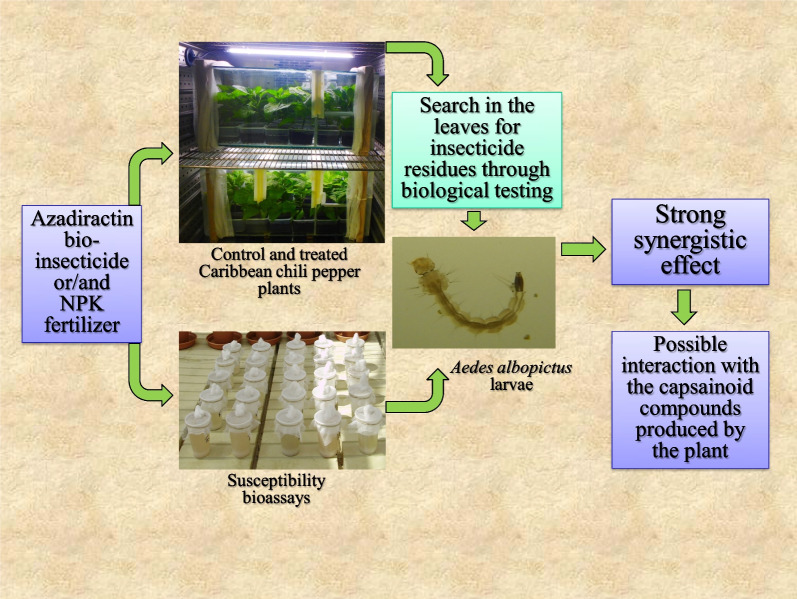

## Introduction

Since the mid twentieth century, intensive farming has been using more and more chemical fertilizers and pesticides to increase crop yields and protect plants from pests. 180 millions tons of fertilizers and 2.4 million tons of pesticides are spread every year worldwide [1]. Modern fertilizers such as NPK are associations of minerals (N = nitrogen; P = phosphorous; K = potassium) meant to provide the plants with additional nutrients needed for their growth. Most of the time they are added to the soil but can also to borne by irrigated waters or sprayed straight onto the foliage [2]. The major parts of the insecticides used in agriculture are sprayed on the aerial parts of the plants. Irrigated crops such as rice and market gardening, besides their own pests, also host some mosquito species whose females inoculate vector-borne diseases to men, such as malaria, Japanese encephalitis, lymphatic filariasis and West Nile virus. Field observations and laboratory studies have confirmed that fertilizers do attract the mosquitoes looking for breeding places [3–6]. Fertilizers are not directly assimilated by the mosquito larvae, however the three minerals [nitrogen, phosphorous, and potassium] enhance the development of bacteria, algae, and fungi, increasing the food biomass of the breeding sites [7]. Mosquito larvae exploit this additional biomass to proliferate [8, 9]. To limit the proliferation of mosquitoes and destructive insects dependent on irrigated crops, an innovative control strategy was implemented in laboratory, consisting of adding to the fertilizer spread on the fields, chemical or biological insecticides (AGRIAC concept) [10]. During the laboratory trial of this novel concept of control, the chemical insecticides, thiacloprid (neonicotinoids family) and cyromazine (aminotriazins family) and the bio-insecticide azadirachtin (limonoids family) have been tested on Caribbean chili pepper plants (*Capsicum chinense*) (Solanales: Solanaceae). Thiacloprid and cyromazine have a systemic action, and azadirachtin, a translaminar action. A systemic insecticide is a substance which gets into the plant so as to be sap-borne while a translaminar compound is a molecule which can penetrate only a few layers of the cells of the same plant organ [11]. A search for insecticide residues in chili pepper plants using biological testing revealed that dried samples from untreated plants displayed natural toxicity (45% mortality) towards *Aedes albopictus* larvae (Diptera: Culicidae). The search for residues also revealed that thiacloprid, cyromazine and azadirachtin show greater insecticide efficacy (susceptibility tests *versus* insecticide residues bioassays) once absorbed by *C. chinensis*. These plants, which belong to the Solanaceae family, synthesize capsaicinoid components (capsaicin and dihydrocapsaicin) in their leaves and fruits that are an irritant to mammals, including humans [12]. To estimate the capsaicin content in the matured fruits of *C. chinensis* (Umorok cultivar), analyses carried out by high performance liquid chromatography (HPLC) revealed an average content of capsaicin of 2.06% (% w/w) [13]. When thiacloprid, cyromazine and azadirachtin insecticides enter *C. chinensis* plants (systemic or translaminar effects), their own toxicity probably adds up to the natural toxicity of the capsaicinoid compounds they come across [10]. Moreover, the bioassays have shown that the synergistic effect of the insecticides with the capsaicinoid compounds is quite substantial with azadirachtin [10]. In order to better encompass these synergistic effects inside the pepper plants, further evaluation was performed on the azadirachtin bio-insecticide alone and/or in combination with an NPK fertilizer. Extracted from the neem tree seeds (*Azadirachta indica*) (Sapindales: Meliaceae), this natural insecticide is used in market gardening to eliminate the phytophagous caterpillars and the piercing and sucking insects feeding on the plant sap [14]. The susceptibility tests were carried out with *Ae. albopictus* larvae so as to measure the activity of the various substances studied (azadirachtin, fertilizer and capsaicin), alone or in combination. The mortalities recorded in these insecticide susceptibility tests were compared with the mortalities induced by the dried plant leaves which had previously received water, NPK fertilizer, azadirachtin or azadirachtin + NPK combination.

## Results and discussion

### Search in chili pepper plants for insecticide residues through biological testing

#### Weight of dried plant leaves and search for insecticide residues (Table 1)

**Table 1:** Averages of weight of dried plant leaves and search for insecticide residues in chili pepper plants through biological testing

	Averages of weight (mg) of dried plant leaves (95% CI)	*P*	NPK or/and azadirachtin quantity in mg^a^	NPK or/and azadirachtin quantity for 100 mg of dried plant/100 ml of water	NPK or/and azadirachtin concentrations in mg/l	N^c^	% Inhibition of adult mosquito emergence (95% CI)
Negative control	–	–	–	–	–	252	2.4 (0.93–3.9)
Water (positive control)	199.2 (125.9–272.5)	0.56	–	–	–	250	29.2 (21.7–36.7)
NPK	262.0 (188.7–335.3)		0.543^b^	0.207	2.07	250	33.6 (26.6–41.4)
Azadirachtin	214.0 (140.7–287.3)		0.054	0.025	0.25	250	36.4 (31.1–41.7)
Azadirachtin + NPK	247.2 (173.9–320.5)		0.054 + 0.543^b^	0.0218 + 0.220	0.218 + 2.20	250	74.4 (53.8–95.0)

The inhibition percentage of *Aedes albopictus* adult emergence (95% CI) was assessed on 100 mg of dried plant in 100 ml of osmotic water for the experimental conditions: water (positive control), NPK fertilizer, azadirachtin insecticide alone and NPK + azadirachtin combination. The negative control consisted of bioassays carried out without dried chili pepper plants. The statistical analysis was performed using the one-way ANOVA test comparing the weight averages (Gaussian distribution) and the Kruskal Wallis test comparing the inhibition percentages of adult mosquito emergence (non-Gaussian distribution)

^a^For the three foliar treatments

^b^This NPK concentration corresponding to 0.21 mg of nitrogen (N) + 0.123 mg of phosphorous (P) + 0.21 mg of potassium (K)

^c^Number of mosquito larvae tested

Whatever the control conditions (negative and positive controls) and treated ones (NPK, azadirachtin and azadirachtin + NPK), the average of weight of dried plant leaves showed no significant differences (P = 0.56). Considering the NPK and azadirachtin doses sprayed on the leaves, the average weight of those leaves in each experimental condition, and the amount selected (100 mg) tested in 100 ml osmotic water, the concentrations of the two agricultural inputs can thus be expressed in mg per liter of water (mg/l). The concentrations in active ingredients amount to 2.07 mg/l for NPK fertilizer, 0.25 mg/l for azadirachtin and 0.218 + 2.20 mg/l for azadirachtin + NPK combination. The negative control (water without plant matter) showed 2.4% larval mortality. Dried plant leaves from the water (positive control), NPK fertilizer and azadirachtin insecticide had a moderate level of toxicity on *Ae. albopictus* larvae with 29.2%, 33.6% and 36.4% mortalities, respectively. If the water, NPK and azadirachtin conditions do not generate significantly different mortalities between them (0.22 < *P* < 0.55), they nonetheless are statistically different from the negative control (*P* = 0.008). A sample from chili pepper plants treated previously with azadirachtin + NPK combination showed much higher toxicity with a mortality rate of 74.4% (NPK + azadirachtin versus other experimental situations: *P* < 0.008).

#### Observed and expected mortalities of NPK + azadirachtin combination (Table 2)

**Table 2:** Activity of NPK + azadirachtin combination (observed and expected mortalities) against susceptible strain IRD *Aedes albopictus*

	% Inhibition of adult mosquito emergence Observed	% Inhibition of adult mosquito emergence Expected	*P* ^a^
Azadirachtin	36.4	–	–
NPK	33.6	–	–
Azadirachtin + NPK	74.4	57.8	0.0001

The statistical comparison between observed and expected mortalities was achieved using a Fisher exact test

^a^*P* < 0.05 = synergistic or antagonistic effect; > 0.05 = additive effect

Compared with the action of azadirachtin and NPK fertilizer alone, the azadirachtin + NPK combination showed a synergistic insecticidal effect against susceptible *Aedes albopictus* (*P* = 0.0001).

### Results of the susceptibility tests

#### Susceptibility tests of capsaicin compound and NPK fertilizer (Table 3)

**Table 3:** Comparative toxicity of capsaicin compound and NPK fertilizer against susceptible strain IRD *Aedes albopictus* tested at the larval stage

	Concentrations in mg/l	% Inhibition of adult mosquito emergence (IC 95%)	N^**a**^
Control	–	10.7 (0.74–20.6)	300
Capsaicin	1	19.0 (6.8–31.2)	800
	3	15.5 (3.3–27.7)	
	5	17.0 (4.8–29.2)	
	10	38.5 (26.3–50.7)	
NPK	5.43	20.7 (0.5–21.8)	300

^a^Number of mosquito larvae tested

Capsaicin showed little toxicity on *Ae. albopictus*, the 1 mg/l to 10 mg/l concentrations causing a mere 19% to 38.5% mortality among the larvae. However the 1 mg/l concentration of capsaicin inducing 19% larval mortality was retained to conduct all of the other tests. As a matter of fact, at the concentration of 5.43 mg/l, the NPK fertilizer induces a very limited lethal response with only 20.7% of dead larvae.

#### Susceptibility tests of azadirachtin alone, azadirachtin + capsaicin and azadirachtin + capsaicin + NPK combinations (Table 4)

**Table 4:** Comparative toxicity of azadirachtin alone, azadirachtin + capsaicin and azadirachtin + capsaicin + NPK fertilizer combinations against susceptible strain IRD *Aedes albopictus* tested at the larval stage

	N^a^	Slope (± SE)	IE_30_ (95% CI)	IE_40_ (95% CI)	IE_50_ (95% CI)	IE_70_ (95% CI)	IE_95_ (95% CI)	*P* ^b^
Azadiractin	1800	5.1 (± 0.5)	1.04 (0.92–1.17)	1.17 (1.05–1.30)	1.31 (1.20–1.44)	1.67 (1.53–1.80)	2.75 (2.41–3.15)	0.019
Azadirachtin + capsaicin (1 mg/l)	1400	4.5 (± 0.5)	0.68 (0.57–0.80)	0.80 (0.68–0.89)	0.89 (0.79–0.10)	1.16 (1.05–1.28)	2.06 (1.75–2.43)	0.035
Azadirachtin + capsaicin (1 mg/l) + NPK (5.43 mg/l)	1800	3.2 (± 0.2)	0.37 (0.32–042)	0.45 (0.40–0.51)	0.54 (0.48–0.60)	0.79 (0.73–0.85)	1.76 (1.58–2.01)	0.49

The percentages inhibition of adult mosquito emergence (IE_30_, IE_40_, IE_50_, IE_70_ and IE_95_) are expressed in mg/l

^a^Number of mosquito larvae tested

^b^*P* values of regression lines

For the azadirachtin alone, the IE_50_ value was 1.31 mg/l. The IE_50_ value of azadirachtin + capsaicin was 0.89 mg/l, indicating that combination activity was 1.5 times as high as azadirachtin alone. Azadirachtin + capsaicin + NPK mixture issues a 0.54 mg/l IE_50_. The latter combination proves 2.4 times as high a larvicidal action as azadirachtin alone.

### Comparison of mortalities recorded in dried plant leaves versus susceptibility tests (Tables 1 and 4)

In 100 mg of dried chili pepper leaves brewed in 100 ml of osmotic water, the concentration of azadirachtin is estimated at 0.25 mg/l. This residual load in plants kills 36.4% of *Ae albopictus* larvae (Table 1). To get similar mortality rates (IE_40_) in the susceptibility tests, the concentrations of azadirachtin alone or combined with capsaicin have to be 1.17 and 0.8 mg/l (Table 4). The capsaicin naturally produced by the chili pepper plants thus increases 4.7 fold the toxic action of azadirachtin (1.17 mg/l versus 0.25 mg/l).

The azadirachtin + NPK combination recorded in 100 mg of dried chili pepper leaves (0.218 + 2.20 mg/l) induced a 74.4% larval mortality (Table 1). To reach the same levels of mortality in the susceptibility tests (IE_70_), the concentration of azadirachtin has to equal 0.79 mg/l (for the azadirachtin + capsaicin + NPK combination) (Table 4). The azadirachtin + NPK treatment directly applied onto the chili pepper leaves has demonstrated an insecticidal action 3.6 times as important as the same combination evaluated during the susceptibility tests (0.218 mg/l versus 0.79 mg/l).

### An innovative approach for the research of xenobiotic substances

The results obtained during the susceptibility tests reveal that an NPK fertilizer added to azadirachtin + capsaicin combination increases the toxicity of the latter on the *Ae. albopictus* larvae, the more so when both the azadirachtin and NPK are absorbed by Caribbean chili pepper leaves. This synergistic phenomenon inside the plant may be accounted for by fact the azadirachtin + NPK combination interacts with the capsaicinoid compounds naturally synthesized by the plant. A similar action had been observed with the thiacloprid and cyromazine insecticides (neonicotinoids and aminotriazins families respectively) also combined with an NPK fertilizer [10]. In this study the content of capsaicin as such in the leaves of *C. chinensis* could not be assessed. However the bioassays carried out on the larvae of *Ae. albopictus* indirectly, yet undoubtedly, testify that once the azadirachtin + NPK combination is sprayed then absorbed by the plant leaves, the latter proves a lot more toxic than each substance separately. If the results of this study carried out with azadirachtin raises interest, even more so the novel approach which discloses the potential to be expected from the interaction with the agricultural inputs within the plants. The chili peppers synthesize capsaicinoid compounds (alkaloids family) but plenty of the other plants worldwide also produce many various similar compounds [15, 16]. Knowing organic molecules from plants can interact with agricultural fertilizer and pesticides to produce xenobiotic compounds even more toxic than the original substances reveals interesting perspectives of research on the isolation and characterization of new substances applicable to agriculture and/or public health. As resistance phenomenons among insects harmful to humans are spreading and intensifying, to find substances with novel modes of action has become urgent, all the more so as the chemical industry has a harder and harder time finding new pesticides families.

## Material and methods

### The biological material

The plant used was the Caribbean chili pepper *Capsicum chinensis* (seeds produced by TECHNISEM). This chili pepper originated from South America is very hot. On the Scoville scale, it scores from 100,000 to 350,000 depending on the variety. For capsaicin [8-méthyl-N-vanillyl-6-nonenamide (alkaloids family)] it scores zero on the same scale, which means no burning sensation is detected, even without dilution. For the *C. chinensis*, the Scoville scale score of 100,000 to 350,000 means that extracts have to be diluted 100,000 to 350,000 times before capsaicin becomes undetectable [17]. By way of an example, the Umorok cultivar of *C. chinensis* has shown that the capsaicin content in the fruits reaches 2.06% (% w/w) with a score of 320,100 on the Scoville scale [13]. All bioassays were conducted with the insecticide-susceptible (SS) tiger mosquito *Aedes Albopictus* IRD strain, raised at the insectarium of the Institute of Research for Development (IRD) (Vectopole) in Montpellier, France.

### The NPK fertilizer, azadirachtin insecticide and capsaicin compound

The 0.058-0.034-0.058 composition of NPK fertilizer (liquid formulation) (Masso Garden) contains 0.058% total nitrogen (N) with 0.0135% ureic nitrogen, 0.0275% ammonium nitrogen (NH_4_ +), and 0.017% nitric nitrogen (NO_3_ −); 0.034% phosphorous (P) in the form of phosphoric anhydride (P_2_O_5_) and 0.058% potassium (K) in the form of potassium oxide (K_2_O). The bio-insecticide azadirachtin (Neemazal®), is presented as a suspension concentrate [EC] at the dosage of 9.8 g/l (Andermatt). The capsaicin compound, analytical standard, (Pestanal™), titrates 98.5% of technical grade (Sigma-Aldrich).

### The experimental design

**Fig. 1:**
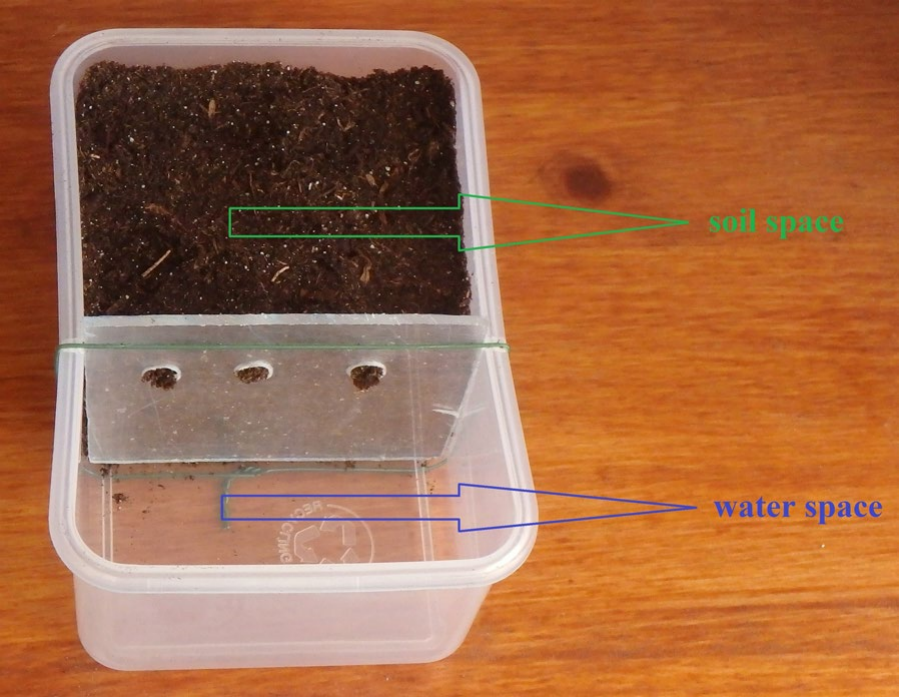
Experimental device used to search for xenobiotics substances in plants (©Frédéric Darriet)

NPK fertilizer or/and the bio-insecticide azadirachtin were evaluated in plastic containers [L: 0.115 m; l: 0.08 m; H: 0.055 m; S: 0.0092 m^2^] two thirds of their surface representing the soil space (0.00616 m^2^). The remaining third of the surface (0.00307 m^2^) was allotted to the water supply needed for the normal development of the plant (water space). The soil and water spaces were separated by a plexiglass sheet with three holes for the water space to communicate with the soil space (Fig. 1). The Caribbean chili peppers seedlings were transplanted in the soil space once they would number about fifteen fully-formed leaves.

### The treatments of Caribbean chili pepper plants

The experiment consisted in evaluating azadirachtin and NPK fertilizer alone and in combination. The treatments were made starting from the dosages applicable for agriculture use of fertilizer and insecticide. The recommended dosage of azadirachtin on market gardening is 30 g/ha, to be reiterated 3 times every 7 days [18]. In foliar spraying, the optimal amount of NPK solution to be sprayed is 200 l/ha [19]. The treated conditions were evaluated in parallel with the water situation in five replicates. The fertilizer and insecticide doses were calculated in relation to the surface of the soil space (0.00616 m^2^) (Table 5). Water, NPK, azadirachtin or azadirachtin + NPK were sprayed three times at weekly intervals. The sprayings were performed on the plant leaves with a small pressure sprayer (https://www.massogarden.com/fr/produits/produit/engrais-foliaire-plantes-d-interieur-spray-pret-a-l-emploi-250ml).

**Table 5:** Quantities of azadirachtin insecticide or/and NPK fertilizer poured in a foliar treatment

	Concentrations	
	mg/application	mg/three applications
Azadirachtin	0.018	0.054
N	0.070	0.21
P	0.041	0.123
K	0.070	0.21

### Search in chili pepper plants for insecticide residues through biological testing

One week after the first treatment, all the leaves of the chili pepper plants of each experimental condition (water – NPK – azadirachtin – azadirachtin + NPK) were slipped between two sheets of paper and left to dry for 15 days at room temperature. The dried-up leaves of each plant were weighed, following which a precise quantity of 100 mg of dried leaves was put into 100 ml of osmotic water (macerate titrated to 1 g/l). A negative control condition with no dried plant was assessed following the same experimental conditions. After 24 h and in each condition, 50 *Ae. albopictus* 2nd instar larvae were introduced, fed with aquarium shrimp food and monitored until adult emergence. The temperature was maintained at 27 °C throughout the test.

### The susceptibility tests

Susceptibility tests on 2nd instar larvae of *Ae. albopictus* were carried out with azadirachtin, capsaicin, azadirachtin + capsaicin and azadirachtin + capsaicin + NPK fertilizer. Groups of 50 larvae were placed in 99 ml of osmotic water completed by 0.1 to 1 ml of azadirachtin insecticide or/and capsaicin and NPK fertilizer (100 ml per plastic cup). Larval bioassays were carried out with technical material (capsaicin) or formulations (azadirachtin and fertilizer). The technical grade of capsaicin was diluted in absolute alcohol and the formulation of azadirachtin and fertilizer in osmotic water. The concentration of capsaicin dropped in each cup was determined according to preliminary bioassays determining the sub lethal concentrations [20% of inhibition of mosquito adult emergence (IE)]. The concentration of NPK put into each cup containing 100 ml of water was 0.543 mg (N: 0.21 mg + P: 0.123 mg + K: 0.21 mg). It corresponds to the among of fertilizer administered in foliar spraying on each chili pepper plant. Translated in mg/l of water, the NPK concentrations respectively amount to 2.1 mg/l, 1.23 mg/l and 2.1 mg/l (5.43 mg/l). The capsaicin compound and the NPK fertilizer were put, separately, in the azadirachtin susceptibility tests. Two plastic cups per concentration and a minimum of 4 concentrations per replicate, providing mortality within a range of 01–99%, were used for each replicate. Each bioassay was done in four to six replicates. The larvae were fed with aquarium shrimp food and monitored until adult emergence. The temperature was maintained at 27 °C throughout the test.

### Statistical analyses

The statistical comparisons were performed with Statistica V10 software [20]. When the experimental situations respond to a Gaussian distribution (averages of weight of dried plants leaves), the statistical comparisons were analyzed using the one-way ANOVA test. When the experimental situations didn’t show a Gaussian distribution (% inhibition of mosquito adult emergence), the Kruskal Wallis test was used. The expected mortality [21] for the azadirachtin + NPK fertilizer mixture was calculated by 1 – (frequency of adults surviving on azadirachtin) × (frequency of adults surviving on NPK fertilizer). It is considered to be synergy (positive interaction) when the observed mortality is significantly higher (Fisher’s exact test) than the expected mortality. The insecticide susceptibility tests were analyzed according to the method of Finney [22] by using Probit® software [23]. This software uses an iterative method of maximum likelihood to fit a regression line between the logarithm of concentration and the probit inhibition of mosquito adult emergence (IE). This software then provided an estimation of IE_1_-_99_ with their 95% confidence intervals.
